# Wavelet-Based Semblance Methods to Enhance the Single-Trial Detection of Event-Related Potentials for a BCI Spelling System

**DOI:** 10.1155/2019/8432953

**Published:** 2019-08-26

**Authors:** Carolina Saavedra, Rodrigo Salas, Laurent Bougrain

**Affiliations:** ^1^Escuela de Ingeniería C. Biomédica, Universidad de Valparaíso, Valparaíso, Chile; ^2^Université de Lorraine, CNRS, INRIA, LORIA, F-54000 Nancy, France; ^3^Centro de Investigación y Desarrollo en Ingeniería en Salud, CINGS, Universidad de Valparaíso, Valparaíso, Chile

## Abstract

Based on similarity measures in the wavelet domain under a multichannel EEG setting, two new methods are developed for single-trial event-related potential (ERP) detection. The first method, named “multichannel EEG thresholding by similarity” (METS), simultaneously denoises all of the information recorded by the channels. The second approach, named “semblance-based ERP window selection” (SEWS), presents two versions to automatically localize the ERP in time for each subject to reduce the time window to be analysed by removing useless features. We empirically show that when these methods are used independently, they are suitable for ERP denoising and feature extraction. Meanwhile, the combination of both methods obtains better results compared to using them independently. The denoising algorithm was compared with classic thresholding methods based on wavelets and was found to obtain better results, which shows its suitability for ERP processing. The combination of the two algorithms for denoising the signals and selecting the time window has been compared to xDAWN, which is an efficient algorithm to enhance ERPs. We conclude that our wavelet-based semblance method performs better than xDAWN for single-trial detection in the presence of artifacts or noise.

## 1. Introduction

Brain-computer interface (BCI) research endeavours to provide new ways of communication for severely handicapped people by translating their brain activity into commands that can be used in a computer or other devices, without using the standard peripheral nerves and muscular pathways. In particular, brain-computer interfaces are control and communication systems that are designed to assist people with motor disabilities, such as people suffering from amyotrophic lateral sclerosis (ALS), spinal cord injury, multiple sclerosis, muscular dystrophies, and cerebral palsy. In this paper, we will focus on the noninvasive BCI [1].

Electroencephalography (EEG) is a noninvasive way of measuring over the scalp the electrical activity occurring as the product of the interactions of neurons in the brain [2]. EEG recordings are usually overlapped with noise and artifacts such as muscle activity. Their presence hinders EEG detection and requires novel methods to remove them from the underlying true brain signal. One important assumption about noise is that it is supposed to be independent of brain activity. It is assumed that brain signals are (almost) instantaneously recorded by the electrodes, implying that each recorded channel is highly correlated with the others. Accordingly, it is possible to conclude those signal components that are not correlated over channels are assumed to be noise or artifacts and can be removed from the recorded signals. A comprehensive survey of signal-processing algorithms for EEG applied to the BCI can be found in [3].

Event-related potentials (ERPs) are neural activities generated involuntarily as a consequence of the occurrence of an expected but rare event. A deflection appears in the EEG signal with a specific polarization and latency. For example, P300 is a cognitive ERP with a positive peak at 300 ms after the stimulus. ERP can potentially be detected with signal-processing techniques and used as a control command in BCI applications, such as the very well-known P300 speller proposed by Farwell and Donchin [4].

Unfortunately, major obstacles still exist in the use of EEG for brain-computer interfaces: signal changes to be detected are very small and high noise such as the signal depletion due to the skull or muscular artifacts is present. To enhance and detect the ERP response, it is necessary to repeat the stimuli and average the responses; however, this reduces the information transfer rate. Several efforts to reduce the number of averaged trials or to directly perform single-trial ERP detection have been made [5–8]. The intention of improving the single-trial classification performance ranges from increasing the database, adding artificial trials [9], where original data are deformed to create new artificial patterns, to EEG source reconstruction or transfer learning methods, where data from multiple users are used to train classifiers to improve the BCI system [10, 11]. The single-trial approach saves time, but the signal-to-noise ratio (SNR) is very low, making ERP detection difficult. On the contrary, when the stimuli are repeated to enhance the ERP detection, these repetitions may become tedious and tiring for the user, and the averaging technique decreases the speed of the spelling. Another problem related to the average of repetitions is the assumption of stationarity. Latency jitter, amplitude variability, or phase artifacts between single trials can cause a flattening up to the elimination of transient characteristics [12, 13].

Usually, in P300-based BCI systems, a temporal window is manually selected [14]. This window is usually chosen to be large enough (within a range of [0, 1] second) to ensure including the ERP components under the study, independently of the user reaction time to the stimulus. However, the ERP responses have different latencies (and amplitudes) for each subject, which comprise irrelevant data to be covered in the temporal window. This can increase both the difficulty of training a classifier with irrelevant variables and the complexity of detecting the ERP. The variance among trials can provide information on the subject's cognitive state, allowing comparisons to be made between subjects [15].

Several studies have shown that it is possible, yet difficult, to distinguish single-trial signals from the EEG background [16–18]. A popular technique is the xDAWN algorithm [19] which automatically enhances the ERP for classification by using spatial filters, combining the multichannel information to put aside useless components. An exhaustive comparative study of several classification techniques is given in [20]. A review of state-of-the-art methods for single-trial detection of event-related potentials can be found in [21]. Finally, a complete survey of the issues that should be considered when designing a new P300-based BCI paradigm can be found in [22].

Wavelets theory has been used in several studies for P300 detection [23–25]. In particular, some advances using wavelets for single-trial detection can be found in [16, 26], where automatic denoising methods are recommended. The fundamental hypothesis of wavelet denoising is that large coefficients correspond to the signal and small coefficients correspond to the noise [27]. The problem with current methods is that they can only denoise one channel at a time, regardless of the information on other channels. This causes them to lose the information provided by the ensemble, such as phase and amplitude information. In ERP studies, the most common mother wavelets that are used are the quadratic B-spline [28–30], the Daubechies wavelets Db4 and Db8 [31], the Symlet wavelet [31, 32], and Coiflet [33]. For example, in [34], a single-trial P300 detection algorithm is presented based on independent component analysis (ICA) and wavelets. Nevertheless, despite these advances, single-trial P300 detection still needs to be improved before it can be made more available for the general public.

In this paper, we introduce a novel method to denoise, localize, and isolate ERPs combining two approaches based on wavelet theory. This formalism is used to study single-trial brain signals based on similarity measures. The first approach simultaneously denoises the signals by using the phase information provided by all the channels in a single trial. Afterward, the second approach combines the phase and the amplitude information of the signals to optimize the time window of the ERP for each user.

The rest of this paper is organized as follows: In Section 2, we presented wavelet theory and semblance analysis to introduce our proposal of using the correlated information of recorded channels to remove noise and automatically establish an appropriate time window for the analysis of each subject. The results are provided in Section 3 and the discussion in Section 4. Finally, our conclusions are given in Section 5.

## 2. Materials and Methods

### 2.1. Wavelet Transforms

The wavelet transform is the inner product of a signal *x*(*t*) with scaled and shifted versions of a *mother waveletψ*(*t*) ∈ *L*^2^(*ℝ*) function [35]. The *continuous wavelet transform* (CWT) uses a continuous wavelet function for the signal analysis:

(1) $$\begin{array}{c} W_{\psi }^{x}\left(a,b\right)={\int^{}}_{-\infty }^{\infty }x\left(t\right)\psi_{a,b}^{*}\left(t\right)dt, \end{array}$$

(2) $$\begin{array}{c} \psi_{a,b}\left(t\right)=\frac{1}{\sqrt{a}}\psi \left(\frac{t-b}{a}\right), \end{array}$$

where *a* and *b* change continuously and *ψ*^*∗*^ is the complex conjugate of *ψ*. The CWT coefficients measure the variation of *x* in a neighborhood of point *b*, whose size is proportional to *a*, obtaining a mapping of a one-dimensional signal into a two-dimensional space.

On the contrary, the *discrete wavelet transform* (DWT) uses filter banks to obtain a multiresolution time-frequency representation. More precisely, the discrete orthogonal wavelet decomposition is obtained using a discretised scale and translation.

### 2.2. Semblance Analysis

Wavelet analysis is also useful for bivariate analysis, making it possible to study two different signals to discover the relationship between them. Cross-wavelet analysis allows us to find the mutual characteristics between signals using the available information in the wavelet transform. The cross-wavelet spectrum [36] of two different signals *x*(*t*) and *y*(*t*) is defined by their wavelet decompositions *W*_*ψ*_^*x*^ and *W*_*ψ*_^*y∗*^ as follows:

(3) $$\begin{array}{c} W_{\psi }^{xy}=W_{\psi }^{x}W_{\psi }^{y*}, \end{array}$$

where *W*_*ψ*_^*xy*^ is a complex value and can be decomposed into amplitude |*W*_*ψ*_^*x*^*W*_*ψ*_^*y∗*^| and phase *θ* = tan^−1^(*ℑ*(*W*_*ψ*_^*xy*^)/*ℜ*(*W*_*ψ*_^*xy*^)).

*Semblance analysis* [37] was introduced to compare two given signals *x*(*t*) and *y*(*t*) based on the phase correlations between their wavelet decompositions *W*_*ψ*_^*x*^ and *W*_*ψ*_^*y*^ using *θ*:

(4) $$\begin{array}{c} S\;=\;\cos^{n}\left(\theta \right),\;S\in \left[-1,1\right], \end{array}$$

where *n* is an odd integer that is greater than zero. The reason why *n* should be odd is to preserve the sign of the cosine. The use of large numbers for *n* also produces a sharp semblance response, as demonstrated in [37].

The values of *S* ∈ [−1,1] correspond to the phase correlation between the two signals, where *S*=1 means they are fully correlated, *S*=−1 means they are fully inversely correlated, and *S*=0 means they are not correlated. Also, it is possible to analyse the signal's amplitudes combining the phase information *S* with the amplitude information as follows:

(5) $$\begin{array}{c} D\;=\;\cos^{n}\left(\theta \right)\left|W_{\psi }^{x}W_{\psi }^{y*}\right|. \end{array}$$

### 2.3. Multisemblance Analysis

The semblance concept was extended to compare more than two signals at the same time. This measure is called the *mean resultant length* (MRL), and it was presented by Cooper in [38] based on circular statistics [39]. The MRL can be calculated according to the number *N* of signals treated, at each scale *a* and time *t*:

(6) $$\begin{array}{c} \text{MRL}\left(a,t\right)=\sqrt{\frac{\sum_{i=1}^{N}ℜ{\left(W_{\psi }^{i}\left(a,t\right)\right)}^{2}+\sum_{i=1}^{N}ℑ{\left(W_{\psi }^{i}\left(a,t\right)\right)}^{2}}{\sum_{i=1}^{N}\left|W_{\psi }^{i}\left(a,t\right)\right|}}. \end{array}$$

For more than two signals, the inversely correlated concept does not apply. This is verified by the MRL values ranging from 0 for uncorrelated signals to 1 for correlated signals.

### 2.4. Denoising Based on the Similarity in the Channels

We propose to denoise EEG signals considering the information of all channels based on their phase and correlations within the DWT transform. Let **X** be the matrix containing the whole dataset, and let *x*_*c*_(*t*) be the signal recorded by the *c*^th^ electrode, *c* ∈ {1,…, *C*}, at time *t*, *t* ∈ {1,…, *T*}. The matrix of the recorded EEG signals can be defined as **X** ∈ *ℝ*^*T*×*C*^. The denoising, through thresholding, can be done using the MRL coefficients, i.e., using coefficients obtained for all channels (equation (6)), instead of the individual channel coefficients. We prune all the coefficients that are below a given threshold *τ*_d_ to zero in order to reconstruct the signal using the filtered wavelet coefficients. The MRL computation is done through the combination of the phase angles of the real and imaginary parts of the wavelet decomposition. DWT uses wavelet families that are orthogonal to each other so that the imaginary part can be achieved by the Hilbert transform of the channel [38].

In simple words, we keep those components with high similarity between channels to produce a denoised EEG signal. This novel approach is called *multichannel EEG thresholding by similarity* (METS), and the full wavelet denoising process is described in Algorithm 1.

### 2.5. Temporal Window Selection

We propose to compute a variable time window, by comparing the averages of the target and nontarget signals. Two alternatives are proposed: by channels or by the grand averages over channels. The purpose is to have a better expressiveness of both amplitude and phase to isolate the ERP wave. The hypothesis is that the ERP is not correlated with the EEG background activity, and this should be reflected in the semblance analysis.

**Figure 1:**
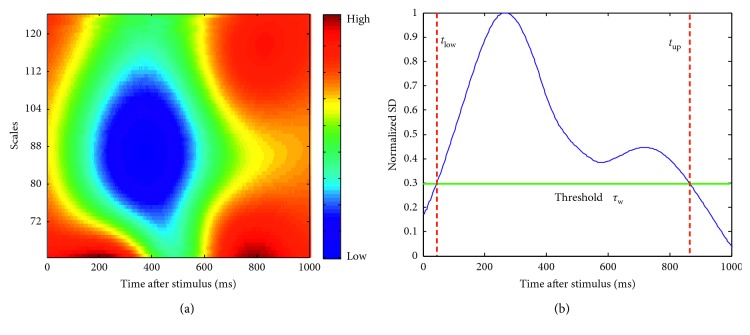
(a) Dot product *D* = cos^*n*^(*θ*)|*W*_*ψ*_^*x*^*W*_*ψ*_^*y∗*^| of the grand average. The colors in the image range from blue to red indicating the similarity between the grand average of target responses *A*^*𝒯*^ and the grand average of nontarget responses *A*^*𝒩*^ based on the amplitude and phase information. (b) Normalised standard deviation of *D* over the scales (between 0 and 1).

Our approach independently computes the correlation between the averages for each channel or the grand averages over channels, using the continuous wavelet transform, to obtain a continuous trend in time of the correlations by each scale. According to Kolev et al. [40], the most significant differences between target and nontarget responses in the frequency domain are found in delta and theta brain rhythms. Consequently, the scales that are used to analyse the signal averages were selected according to these rhythms.  Figure 1(a) presents the dot product obtained by applying equation (5) to the grand average where it is easy to identify the less correlated components in cold colors and the more correlated components in warmer colors. These warmer colors correspond to the EEG background, yet the correlation is not uniform between scales. It is possible to localise the ERP (in blue) within a thinner time window compared to the original. However, the ERP is not present in all scales, making difficult an automatic detection of this temporal window. Fortunately, the ERP produces a high variability over the scales at a specific time on the dot product *D* given by equation (5), which leads to analysis of the standard deviation for each time point. As the magnitudes of the wavelet coefficients can be different for each subject, we normalise the standard deviation between 0 and 1. Finally, we select the temporal window utilising a predefined threshold, as shown in Figure 1(b). This technique is completely independent of the previous denoising step introduced in Section 2.4, although better results are expected if both techniques are used together.

#### 2.5.1. Semblance-Based ERP Window Selection by Channels

Let the signals be denoted by *x*_*c*_(*t*), where *c* corresponds to the channel and *t* ∈ {1,…, *T*}, and *ℳ* be the set of all the stimuli, *ℳ*={*𝒯*, *𝒩*}, in which *𝒯* corresponds to the subset of target stimuli and *𝒩* corresponds to the subset of nontarget stimuli. The responses to a stimulus in a predefined temporal window of size *t*^up^ can then be extracted as follows:

(7) $$\begin{array}{c} r_{i,c}\left(t\right)=x_{c}\left(s_{i}+t\right),\;t\in \left\{1,\ldots ,t^{\text{up}}\right\}, \end{array}$$

where *s*_*i*_ corresponds to the stimulus onset *i*. The average response for each type of stimulus, and for each channel, can be computed as follows:

(8) $$\begin{array}{c} A_{c}^{T}\left(t\right)=\frac{1}{\left|T\right|}\sum_{i\in T}r_{i,c}\left(t\right),\\ A_{c}^{N}\left(t\right)=\frac{1}{\left|N\right|}\sum_{i\in N}r_{i,c}\left(t\right), \end{array}$$

in which the operator |·| denotes the cardinal number. After obtaining the averages, we compute their continuous wavelet transforms *W*_*ψ*_^*A*_*c*_^*𝒯*^^ and *W*_*ψ*_^*A*_*c*_^*𝒩*^^ to calculate the dot product *D* through equation (5).

In the example given in Figure 1, the result obtained for the dot product *D* corresponds to Figure 1(a), where the cold colors indicate that the signals have a maximum difference, and P300 is located in the spatial space. The normalised standard deviation of *D* is shown in Figure 1(b). By using a threshold *τ*_w_, 0 ≤ *τ*_w_ ≤ 1, the new temporal window, for the current channel, is selected within limits *t*^low^ and *t*^up^ obtained through a sequential search of the first points that exceed the threshold from both the left and right boundaries. We named this algorithm “*semblance-based ERP window selection by channels”* (SEWS-1), and Algorithm 2 describes the complete window selection process.

#### 2.5.2. Semblance-Based ERP Window Selection over Channels

A variation of this model is used to perform the *semblance-based ERP window selection over channels* (SEWS-2), as summarised in Algorithm 3, where the same temporal window is selected for all channels. To do this, the only difference in the process is to compute the grand average through all of the channels using

(9) $$\begin{array}{c} GA^{T}\left(t\right)=\frac{1}{\left|C\right|}\overset{C}{\underset{c=1}{\sum }}A_{c}^{T}\left(t\right),\\ GA^{N}\left(t\right)=\frac{1}{\left|C\right|}\overset{C}{\underset{c=1}{\sum }}A_{c}^{N}\left(t\right). \end{array}$$

Further information about algorithms and methods can be found in [41].

### 2.6. Databases

#### 2.6.1. UAM Dataset

We employ a P300 dataset [42] recorded by the Neuroimaging Laboratory of Universidad Autónoma Metropolitana (UAM), Mexico, using the P300 speller [4] included in the BCI2000 platform [43]. The dataset (http://akimpech.izt.uam.mx/p300db/; https://tinyurl.com/ycr52v9b) contains 22 first-time healthy users, and all subjects have similar characteristics, such as sleep duration and age. A total of 10 electrodes were recorded (Fz, C3, Cz, C4, P3, Pz, P4, PO7, PO8, and Oz) providing the best features for discrimination [20, 44]. The signals were recorded at 256 Hz using a g.tec g.USBamp EEG amplifier, a right ear reference, and a right mastoid ground. An 8th order 0.1–60 Hz Chebyshev bandpass filter and a 60 Hz notch were used. The stimulus is highlighted for 62.5 ms with an interstimulus interval of 125 ms. In order to validate our algorithms, we use session 1 (copy spelling session) to train a classifier and session 3 (free spelling session) to test the generated models. This choice was made because both sessions do not use feedback and because the detection methods have to be robust under different conditions; that is, the algorithms will be studied under a multisession scheme. From a single-trial perspective, the dataset contains 240 letters per person for session 1 and 250 letters approximately for session 3 because the number of letters varies depending on the subject. Finally, considering that, for the detection of one letter, it is necessary to identify two P300s, i.e., detecting twice 1 ERP among 6 responses, and the letter detection by chance is 1/36.

#### 2.6.2. EPFL Dataset

This dataset (for further details about subjects, see http://mmspg.epfl.ch/BCI_datasets) [45] was recorded by the Ecole Polytechnique Fédérale de Lausanne utilising six different images to evoke a P300 response. The images were individually and randomly flashed for 100 ms with an interstimulus interval of 400 ms. 32 electrodes were recorded (Fp1, AF3, F7, F3, FC1, FC5, T7, C3, CP1, CP5, P7, P3, Pz, PO3, O1, Oz, O2, PO4, P4, P8, CP6, CP2, C4, T8, FC6, FC2, F4, F8, AF4, Fp2, Fz, and Cz) using a BioSemi ActiveTwo system at a sampling rate of 2048 Hz.

## 3. Results

This section presents the experiments for evaluating the performance of the new methods, which are introduced by contrasting them with the xDAWN algorithm and the state of the art on wavelet thresholding techniques.

### 3.1. Experiment: METS vs. Classic Wavelet Methods

Table 1 shows the results of our denoising algorithm METS compared with the results of the classic thresholding methods. The baseline results only using a bandpass filter of [0.1–20] Hz are also presented. All thresholding techniques improve the baseline result on average, meaning that these techniques are actually denoising the original signals and they do not remove the relevant information. We can observe that the performance of the subject with the maximum value slightly decreases using METS. However, our algorithm is, in general, able to remove the noise from the signal's subjects. This improves the results obtained by the classic thresholding techniques based on wavelets. Moreover, the standard deviation is less in our approach, meaning that the increase in the mean is global and not due to the improvement of subjects with best results. In fact, the maximum value is the same as that obtained by minimax and universal thresholds, while the minimum is improved by METS.

### 3.2. Experiment: METS vs. SEWS Algorithms

In this section, we apply our two main contributions, the SEWS techniques and the METS denoising algorithm, to analyse the impact of a smart selection of a thinner window.

The time windows for the subjects range within [0–133] ms for the starting point and within [762–1000] ms for the ending point. The mean and standard deviation for the starting point of the window for METS & SEWS-1 are 14.55 ± 17.11 and for METS & SEWS-2 are 26.5 ± 36.05. The mean and standard deviation for the ending point of the window for METS & SEWS-1 are 951.14 ± 31.86 and for METS & SEWS-2 are 917.32 ± 60.12. Finally, the window sizes for METS & SEWS-1 are 936.64 ± 27.12 and for METS & SEWS-2 are 890.82 ± 54.41.

Table 2 shows the impact of using the METS algorithm as the previous step for the time-window selection. The threshold values for the algorithms are *τ*_d_=0.999 for METS, *τ*_w_=0.1 for SEWS-1, and *τ*_w_=0.2 for SEWS-2, according to the previous study [41].

### 3.3. Experiment: Comparison with xDAWN Algorithm

The xDAWN algorithm plays a similar role to the algorithms proposed in this paper because METS also uses the multichannel information.

Table 3 presents the results of xDAWN, METS & SEWS-1, and METS & SEWS-2, for each subject. The minimum sample size for each subject was 2160 trials. Note that the baseline performance is 1/36=2.8% for a random detection. The preprocessing of the signal was performed according to the specifications in [19]. The only difference from our experimental setup is the high cutoff filter frequency of 1 Hz, instead of 0.1 Hz. For xDAWN, three sources (channels) were used because previous studies have shown that the best result was obtained using this configuration [41].

To check the normality assumption, we applied the *Shapiro–Wilk pairwise test* with a significant level *α* of 5%. For the combination of xDAWN with METS & SEWS-1 and xDAWN with METS & SEWS-2, the difference of performances is not normally distributed. On the contrary, for the difference of performances between METS & SEWS-1 with METS & SEWS-2, we cannot reject the normality assumption. Finally, the performance of the METS-SEWS algorithms is statistically greater than that of xDAWN according to the *Wilcoxon signed-rank test* with a significant level *α* of 5%. However, the METS & SEWS-1 and METS & SEWS-2 have no statistically significant difference according to the *t*-test (*α*=5%).

Table 4 summarises the statistics of the letter accuracy for xDAWN compared to our algorithms. Our proposed algorithms obtained the best results, showing improvements in mean, standard deviation, and maximum and minimum values. Surprisingly, the mean results for xDAWN do not improve the baseline of 52.50% (see Table 4) obtained with the [1–19] Hz filter, achieving good results for subjects with high performances and very low results for subjects with low performances, as shown in Table 3.

Table 5 shows the results corresponding to the evolution of the algorithms when using different numbers of repetitions, going from a single trial up to five repetitions. It is possible to observe that METS & SEWS-2 perform better than METS & SEWS-1 using repetitions. As was expected, METS & SEWS perform better than xDAWN for a few (two and three) repetitions. For four repetitions, the results are very similar, and for five repetitions, xDAWN outperforms our algorithms.

The results for the EPFL dataset are reported in Table 6. To check the normality assumption, we applied the *Shapiro–Wilk pairwise test* with a significant level *α* of 5%. For all the combination of algorithms, we cannot reject the normality assumption. The performance of the METS-SEWS algorithms is statistically greater than that of the baseline and the METS algorithms according to the *t-test* with a significant level *α* of 5%. However, METS & SEWS-1 and METS & SEWS-2 have no statistically significant difference according to the *t*-test (*α*=5%). It is important to note that the combination of METS and SEWS offers an improvement for almost all the subjects. Specifically, SEWS-2 is more effective for disabled subjects (i.e., the first four subjects). Moreover, it obtains an impressive improvement from 45.31% to 62.50% for the healthy subject numbered s8.

## 4. Discussion

The results show that the introduction of the channels' correlation in the process and the fact that the channels are processed as a whole improve the denoising step for the classification. The thresholds in classic methods are selected using statistical techniques to infer a suitable threshold for each subject, while for METS, we used a fixed threshold *τ*_d_ for all subjects. Thus, METS could be extended to use a similar automatic threshold selection to obtain better results.

Although the improvements compared to the classical wavelet methods may seem modest in terms of absolute values, we should take into consideration that the probability of detecting a letter in the P300 speller, by chance, is only 1/36 (detecting twice 1 ERP among 6 responses), in contrast to the probability of randomly detecting a single ERP of 1/2. This means that there is a high probability of missing a letter that was correctly detected (0.97) because of randomness and only a small chance of correctly detecting a letter (0.03) that was previously misclassified.

Also, the METS algorithm is flexible enough to exploit the specific properties of the analysed phenomenon through the parameter selection. In fact, the mother wavelet is one important parameter. Unfortunately, there is no formal method to choose a mother wavelet. It can only be chosen by its resemblance in shape to the underlying target phenomenon or by their properties.

Table 3 suggests that the data of subjects with high accuracy are less noisy or have stronger ERP responses, making the classification results similar for all methods. However, for a more general solution that can deal with signals from subjects with poor original performance, either because they are first-time users or because they do not generate a strong P300 response, our approach seems more suitable. Therefore, we conclude that wavelet-based semblance performs better than xDAWN for single-trial detection in the presence of artifacts and noise. Regardless, xDAWN should have a better performance using averaged responses. These results are consistent with the theory behind both algorithms: xDAWN improves with more repetitions because averaged ERPs are easier to enhance due to the increased SNR, while the effect of METS becomes moderate because averaging naturally removes uncorrelated components. Nevertheless, xDAWN and our proposed algorithms are not mutually exclusive, which means that their clever combination could be developed in the future.

As is explained in Section 2.5, a temporal window must be set to specify the segment to be analysed. The removed information usually lies at the end of the original temporal window, beyond the segment where P300 theoretically lies. Both SEWS algorithms improve the results compared to the results of only using METS. When comparing with the baseline filter results, the combination of our two independent algorithms significantly improves the ERP detection in a single trial and only incurs a small computational cost compared to the time required for the training and classification phases. In particular, SEWS-1 obtains the best maximum and minimum accuracy, but, on average, both window selection algorithms are competitive. Besides, a better SNR makes the classification easier and implies that useless features removed by SEWS have less impact on the decision boundaries.

The experiment using the EPFL dataset validates our techniques for single-trial ERP detection not only because it is a different dataset with a different display matrix and patients but also because it uses the same parameters used for the UAM dataset. Finally, better performances are expected if the parameters (like the thresholds) are fine tuned for this dataset.

## 5. Conclusion

We introduced two automatic methods in this paper. The first method aims to simultaneously denoise channel recordings, which allows us to detect signal components that are not present in all channels and improve the analysis of EEG signals in general. The second method uses the continuous wavelet transform to analyse ERP's time windows. The core element of the time-window selection algorithms is the dot product *D*, and the techniques presented in this paper only explore a few of the possibilities of what can be done using *D*. The standard deviation exploits the information carried by *D*. A more elaborate or detailed method could be developed in the future to increase the performance or robustness of the time-window selection. Moreover, our analysis of the similarity of signals based on wavelets opens several research opportunities for EEG applications. For example, the same principle behind the time-window selection can be applied to detect constant scales through the full signal to select the most useful frequency bands for the problem under study.

Finally, these similarity measures can be applied to the study, and comparison of the EEG behavior of different subjects for the same application can be applied to help us better understand the physiological responses of the brain or to develop more robust BCI techniques.

## Figures and Tables

**Algorithm 1:**
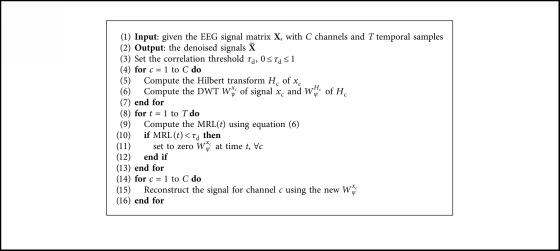
Multichannel EEG thresholding by similarity (METS).

**Algorithm 2:**
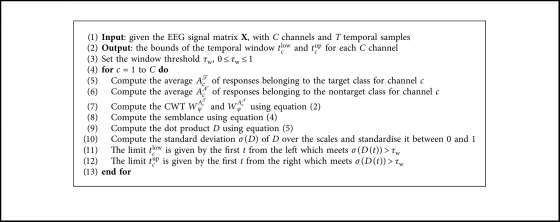
Semblance-based ERP window selection by channels (SEWS-1).

**Algorithm 3:**
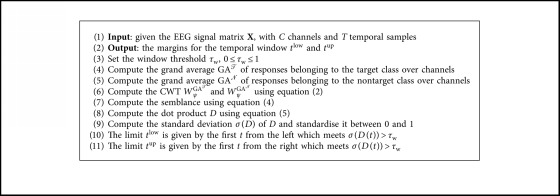
Semblance-based ERP window selection over channels (SEWS-2).

**Table 1:** Letter accuracy for the METS, minimax, universal, and SURE denoising algorithms over 22 subjects of the UAM dataset. Descriptive statistics for all the subjects on letter accuracy are reported (average, standard deviation (SD), min., and max.).

Method	Average	SD	Min.	Max.
[0.1–20] Hz filter	53.60	14.14	28.25	**79.52**
SURE	54.80	13.90	33.02	78.57
Minimax	55.00	13.93	32.70	79.05
Universal	55.07	13.92	33.02	79.05
METS	**55.20**	**13.69**	**33.65**	79.05

The best performances are highlighted in bold.

**Table 2:** Letter accuracy for the time-window selection algorithms (SEWS) when using denoising algorithms (METS), over 22 subjects of the UAM dataset. Descriptive statistics for all the subjects on letter accuracy are reported (mean and standard deviation).

Preprocessing	Average	SD
[0.1–20] Hz filter	53.60	14.14
METS	55.20	**13.19**
METS & SEWS-1	**56.00**	13.64
METS & SEWS-2	55.91	14.13

The best results are highlighted in bold.

**Table 3:** Letter accuracy of single-trial detection for the 22 subjects for xDAWN, METS & SEWS-1, and METS & SEWS-2 algorithms.

Subject	METS & SEWS-1	METS & SEWS-2	xDAWN
s1	**50.00**	49.52	49.05
s2	**66.67**	64.31	60.78
s3	58.75	59.17	**60.42**
s4	35.56	35.56	**36.19**
s5	70.28	**71.39**	71.11
s6	38.15	**40.00**	31.85
s7	**36.67**	34.44	24.44
s8	68.52	**72.59**	67.78
s9	**62.38**	**62.38**	55.24
s10	**44.76**	43.81	**44.76**
s11	**50.88**	48.77	41.40
s12	**61.67**	58.33	56.11
s13	45.56	46.30	**47.78**
s14	52.08	**52.50**	43.75
s15	**46.67**	43.81	28.10
s16	76.41	**78.97**	61.54
s17	**80.00**	78.57	**80.00**
s18	57.68	58.26	**58.55**
s19	74.00	**74.33**	73.67
s20	**39.65**	39.30	36.49
s21	46.67	**47.78**	30.28
s22	68.89	**70.00**	63.33

The best results are highlighted in bold.

**Table 4:** Comparison between METS & SEWS and xDAWN algorithms. Descriptive statistics for all the subjects on letter accuracy are reported (mean, standard deviation, min., and max.).

Preprocessing	*μ*	*σ*	Min.	Max.
[1–19] Hz filter	52.50	13.49	30.48	76.41
xDAWN	51.03	15.80	24.44	80.00
METS & SEWS-1	**56.00**	**13.64**	**35.56**	**80**
METS & SEWS-2	55.91	14.13	34.44	78.97

The best results are highlighted in bold.

**Table 5:** Letter accuracy comparison using the average of trials for xDAWN and METS & SEWS algorithms. The parameters used for METS & SEWS algorithms are the same as in Table 4.

No. of repetitions	1	2	3	4	5
xDAWN	51.03	67.26	72.36	**75.81**	**82.28**
METS & SEWS-1	**56.00**	67.07	72.97	73.10	77.57
METS & SEWS-2	55.91	**68.31**	**73.40**	75.28	78.09

The best results are highlighted in bold.

**Table 6:** P300 detection percentage for the EPFL dataset using the proposed algorithms for each subject. Descriptive statistics for all the subjects on the P300 detection percentage are also reported (mean, standard deviation, min., and max.).

Subject	Baseline	METS	METS & SEWS-1	METS & SEWS-2
s1	44.53	40.88	42.34	**45.26**
s2	41.41	49.22	49.22	**50.00**
s3	58.33	62.88	**64.39**	**64.39**
s4	49.21	48.41	**52.38**	50.00
s5	44.62	46.15	**46.92**	43.08
s6	48.18	54.01	**60.58**	55.47
s7	**72.93**	65.41	70.68	**72.93**
s8	45.31	53.12	55.47	**62.50**
Average	50.57	52.51	55.25	**55.45**
SD	10.36	**8.28**	9.49	10.36
Min.	41.41	40.88	42.34	**43.08**
Max.	**72.93**	65.41	70.68	**72.93**

The best results are highlighted in bold.

## Data Availability

The data used to support the findings of this study are available at http://mmspg.epfl.ch/BCI_datasets and http://akimpech.izt.uam.mx/p300db/.
